# Editorial Note: Comparing public support for nuclear and wind energy in Washington State

**DOI:** 10.1371/journal.pone.0349899

**Published:** 2026-05-28

**Authors:** The PLOS One Editors

The *PLOS One* Editors issue this Editorial Note because this article [1] was identified as one of a series of submissions for which we have concerns about aspects of the peer review process. Readers are advised to interpret the article [1] with caution.

The Editors have no evidence of any author involvement in the peer review concerns.

## References

[1] Uji A, Song J, Dolšak N, Prakash A. Comparing public support for nuclear and wind energy in Washington State. PLoS One. 2023;18(4):e0284208. doi: 10.1371/journal.pone.0284208 37099485 PMC10132544

